# RNA-Seq Reveals Leaf Cuticular Wax-Related Genes in Welsh Onion

**DOI:** 10.1371/journal.pone.0113290

**Published:** 2014-11-21

**Authors:** Qianchun Liu, Changlong Wen, Hong Zhao, Liying Zhang, Jian Wang, Yongqin Wang

**Affiliations:** Beijing Vegetable Research Center, Beijing Academy of Agriculture and Forestry Sciences/Key Laboratory of Biology and Genetic Improvement of Horticultural Crops (North China), Ministry of Agriculture, Beijing 100097, P.R. China; East Carolina University, United States of America

## Abstract

The waxy cuticle plays a very important role in plant resistance to various biotic and abiotic stresses and is an important characteristic of Welsh onions. Two different types of *biangan* Welsh onions (BG) were selected for this study: BG, a wild-type covered by wax, which forms a continuous lipid membrane on its epidermal cells, and GLBG, a glossy mutant of BG whose epidermal cells are not covered by wax. To elucidate the waxy cuticle-related gene expression changes, we used RNA-Seq to compare these two Welsh onion varieties with distinct differences in cuticular wax. The de novo assembly yielded 42,881 putative unigenes, 25.41% of which are longer than 1,000 bp. Among the high-quality unique sequences, 22,289 (52.0%) had at least one significant match to an existing gene model. A total of 798 genes, representing 1.86% of the total putative unigenes, were differentially expressed between these two Welsh onion varieties. The expression patterns of four important unigenes that are related to waxy cuticle biosynthesis were confirmed by RT-qPCR and COG class annotation, which demonstrated that these genes play an important role in defense mechanisms and lipid transport and metabolism. To our knowledge, this study is the first exploration of the Welsh onion waxy cuticle. These results may help to reveal the molecular mechanisms underlying the waxy cuticle and will be useful for waxy gene cloning, genetics and breeding as well as phylogenetic and evolutionary studies of the Welsh onion.

## Introduction

The Welsh onion (*Allium fistulosum* L.) is a perennial herb that is widely cultivated worldwide, from tropical Asia to China, Korea, Japan and as far north as Siberia [1]. The Welsh onion is rich in carbohydrates, proteins, mineral salts and vitamins and contains propylene sulfide, which has bactericidal and anti-inflammatory effects. The Welsh onion has been used as an herbal medicine for many diseases by activating immunity, preventing colds, and treating febrile disease, headache, diarrhea, abdominal pain, eye-related disorders, and habitual abortion [2]. Currently, the research on the Welsh onion has focused on increasing the yield [3]–[5], the mechanisms of freezing resistance [6], the resistance to *Fusarium oxysporum* in *Allium fistulosum* [7],[8], the extraction of medicinal ingredients [9],[10], and reducing the pungency of agronomic traits [11].

The outermost layer of the Welsh onion is composed of the cuticle and an outer epidermis of wax. The cuticular wax consists of derivatives of long-chain fatty acids, including alkanes, primary alcohols, secondary alcohols, aliphatic aldehydes, ketones and esters [12]. The cuticular wax can protect tender leaves against insects or other abiotic stresses, including moisture loss [13], bacteria and herbivorous insects [14], and the effects of ultraviolet radiation and frost damage [15]–[17].

The wax is mainly composed of very-long-chain fatty acids (VLCFAs) in the plant cuticle, with the fatty acids having a carbon number of 18 or higher. Such fatty acids have a wide range of physiological functions; are involved in the synthesis of seed glycerides, sphingolipids and lipid biofilms; and provide precursors for the biosynthesis of the waxy cuticle. As the waxy cuticle plays an important role in the plant's resistance to adversity, researchers are focusing on understanding the waxy synthesis pathway, gene cloning and functional characterization [18].

Several wax-related genes were isolated in maize and the model plant *Arabidopsis*; *CER6*, *CER10, GL8A, GL8B, FDH, FAE1, KCS1* and *PAS2* are involved in the synthesis of VLCFA wax precursors [19]–[27]. *CER4* and *WSD1* participate in the acyl reduction pathway to catalyze the production of primary alcohol and wax ester, respectively, and are involved in the synthesis of wax components [28]–[29]. *MAH1* participates in the decarbonylation pathway to catalyze the conversion of alkanes into secondary alcohols and ketones [30]. *CER5* in *Arabidopsis* is the first characterized gene that encodes the plasma membrane-localized ABC transporter that is required for the transport of wax components from the epidermal cells to the cuticle [31]. Our study demonstrates that four Welsh onion unigenes that are related to waxy cuticle synthesis function in the several processes, including long-chain fatty acid metabolism, very-long-chain fatty acid metabolism, wax biosynthesis, and oxidation-reduction; therefore, these genes may be involved in the synthesis of wax precursors.

Welsh onion is a typical waxy plant, but the wax content and genes are not well studied. This study was designed to compare the RNA-seq results between waxy plants and non-waxy mutant plants via high-throughput sequencing technology and to identify the important genes that play key roles in wax synthesis in the Welsh onion. The high-throughput sequencing results will also reveal other pathways that are related to wax synthesis and will identify a larger number of polymorphism molecular markers (SSRs and SNPs), which are scarce in the Welsh onion. Based on the RNA-seq data, the closely related gene expression patterns were investigated to illustrate the function of these genes in the wax synthesis pathway. This research will provide additional evidence of waxy gene expression in wax synthesis and can be used to develop methods for mapping waxy genes and other genes in the Welsh onion.

## Materials and Methods

### Sample Preparation

**Figure 1:**
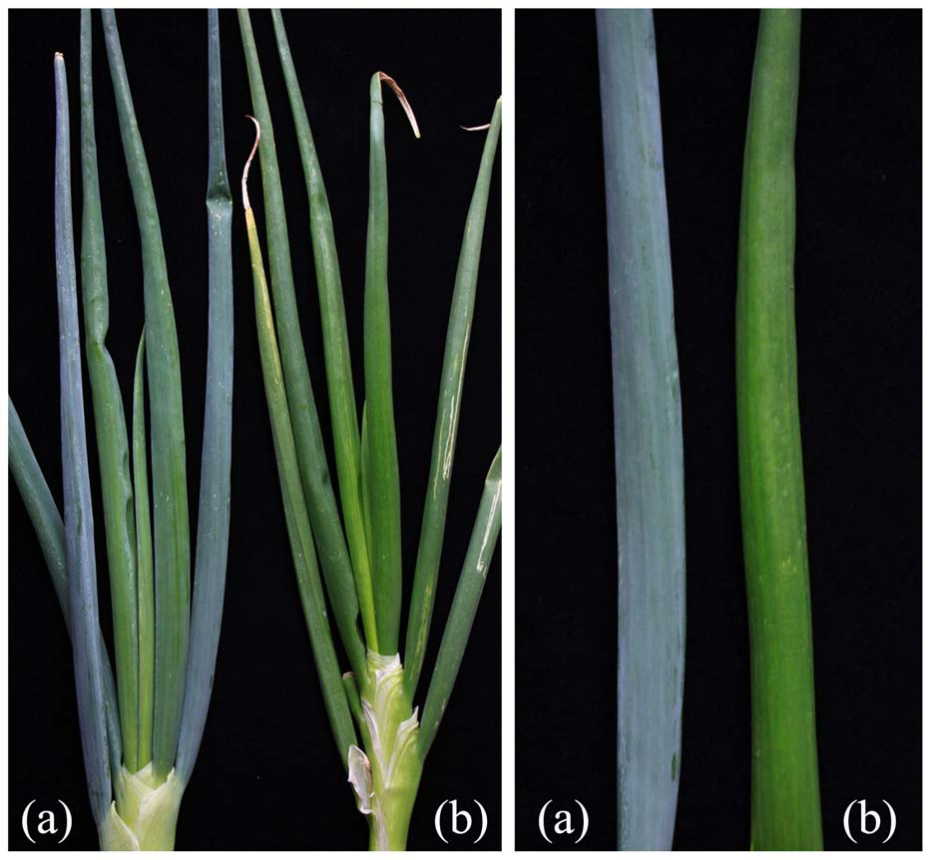
Comparison of the variety covered by wax with that not covered by wax. (a): BG, a wild type whose epidermal cells are covered by wax; (b): GLBG, a variety whose epidermal cells are not covered by wax.

Two BianGan Welsh onion varieties (Chinese accessories) were used in this study, which are BianGan Welsh onion (BG) and glossy BianGan Welsh onion (GLBG) (Figure 1). These two Welsh onion varieties differ in cuticular wax on leaves, The BG Welsh onion was known as waxy Welsh onion, because it was accumulating large amounts of epicuticular wax, resulting in blue gray color in foliage, but the GLBG Welsh onion was known as glossy Welsh onion, it was one mutant derived from BG Welsh onion, which leaves was glossy and bright green. In addition, The waxy BG Welsh onion was tolerant to leaf pathogens, and expressing less transpiration and spray injury, and GLBG Welsh onion was expressing less thrips populations and less feeding damage area compared with BG Welsh onion. Therefore, the waxy foliage was one important trait of BG Welsh onion, and genetic analysis indicated that waxy foliage trait was controlled by one single recessive gene (unpublished date). These materials were bred at the Beijing Academy of Agriculture and Forestry Sciences. For each variety, RNA was isolated from three mature leaves that were picked in November 2013. All of the samples were ground in a mortar in liquid nitrogen immediately after harvest. The total RNA was extracted using an RNAiso for polysaccharide-rich plant tissues kit (TaKaRa Biotechnology, Dalian, Liaoning Province, China). The RNA integrity was evaluated using an Agilent 2100 Bioanalyzer (Agilent Technologies, Santa Clara, CA, USA).

The isolation of mRNA, fragment interruption, cDNA synthesis, addition adapters, PCR amplification and RNA-Seq were performed at Beijing BioMarker Technologies (Beijing, China). The paired-end library preparation and sequencing were performed following standard Illumina methods. The cDNA library was sequenced on the Illumina sequencing platform (HiSeq 2000).

The generated sequence dataset was deposited at National Center for Biotechnology Information (NCBI) in the Short Read Archive (SRA) database under run accession number SRR1609126, SRR1609976.

### De novo Assembly of the Sequencing Reads and Sequence Clustering

To obtain high-quality clean read data for de novo assembly, the raw reads were filtered by removing the adaptor sequences, duplication sequences, reads with an “N” rate greater than 10% (the “N” character representing ambiguous bases in reads), and low-quality reads with more than 50% of the bases with a Q-value ≤5 [32]. The clean reads were assembled into contigs using the Trinity method [33], which efficiently reconstructs full-length transcripts across a broad range of expression levels and sequencing depths. Contigs were created by combining reads that had a certain length of overlap. The reads were then mapped back to the contigs; with paired-end reads itis able to detect contigs from the same transcript as well as the distances between these contigs. The contigs were connected using the Trinity software to get sequences that could not be extended at either end. Such sequences were defined as unigenes, and the unigenes were combined to produce the final assembly used for annotation.

We quantified the transcript levels in reads per kilobase of the exon model per million mapped reads (RPKM). The RPKM measure of read density reflects the molar concentration of a transcript in the starting sample by normalizing the RNA length and the total read number in the measurement [34]. By using Bowtie [35], each sequencing read sample was compared with the UniGene database and, using RPKM, reflected the expression abundance of the unigenes [36].

### Detection of SSR Markers

SSRs were detected among the unigenes that were longer than 1 kb using the software MISA (MIcroSAtellite identification tool; http://pgrc.ipk-gatersleben.de/misa) [37]. A total of 6 types of SSRs were investigated, including mono-, di-, tri-, tetra- and penta-nucleotide repeats and the compound SSR (the sequence contains two adjacent distinct SSRs that are separated by any number of base pairs, including zero).

### Functional Annotation

The unigenes were aligned with the non-redundant (Nr) protein and nucleotide (Nt) databases, the Swiss-Prot protein database, the Kyoto Encyclopedia of Genes and Genomes (KEGG) pathway database, and the Cluster of Orthologous Groups (COG) database. The Gene Ontology (GO; www.gene-ontology.org) is a standardized gene functional classification system that offers a dynamic-updated controlled vocabulary and a strictly defined concept to comprehensively describe properties of genes and their products in any organism. GO has three ontologies: molecular function, cellular component and biological process. Every GO-term belongs to a type of ontology. For the unigenes that had matches to proteins in the NR database. Blast2GO was used to obtain the GO annotation of the unigenes [38]. COG and KEGG pathway annotation were also performed using BLAST against the Cluster of Orthologous Groups databases and Kyoto Encyclopedia of Genes and Genomes. The above searches were performed with a cut-off e-value of 10^−5^.

### Digital Gene Expression Analysis

The IDEG6 software [39] was used to identify differentially expressed genes in a pair-wise comparison, and the results of the statistical tests were corrected for multiple testing with the Benjamini–Hochberg false discovery rate (FDR <0.01). The sequences were deemed significantly differentially expressed if the adjusted P value obtained by this method was <0.001 and if there was at least a twofold change (>1 or <−1 in the log_2_ ratio value) in the RPKM between the two libraries.

### Quantitative RT-PCR (qRT-PCR) Analysis

The cDNA was synthesized from total RNA using PrimeScript II RTase (TaKaRa, Japan) in a 10 µL reaction system. The reverse transcription reaction mixture contained 5 µL of total RNA (0.8 µg), 1 µL of oligo dT (50 µM) (TaKaRa, Japan), 1 µL of 10 mM of each dNTP and 3 µL of DEPC water. The mixture was incubated at 65°C for 5 min and cooled on ice for 5 min, after which 4 µL of 5× PrimeScript II Buffer, 0.5 µL of RNase Inhibitor (40 U/µL), 1 µL of PrimeScript II RTase (200 U/µL) and 4.5 µL of DEPC water were added. The mixture was incubated at 45°C for 1 h. The enzyme was inactivated by incubating at 95°C for 5 min. The qPCR was carried out on the LightCycler 480 II Real-Time PCR Detection System (Bio-Rad, USA) with SYBR Green I (TaKaRa, Japan). The primers used in the qPCR to validate the differentially expressed genes are shown in Table S2. Each gene was analyzed in triplicate, after which the average threshold cycle (CT) was calculated per sample, and an endogenous *actin* gene was used for normalization. The relative fold changes in gene expression were calculated using the comparative Ct (2^−ΔΔCt^) method.

## Results

### Sequence Analysis and Assembly

An Illumina HiSeq 2000 system was used for RNA-seq, and to maximize the range of transcript diversity, a mixed RNA tissue sample from three leaves was used. After stringent quality assessment and data filtering, a total of 47.30 million read pairs corresponded to 9.55 Gb of sequence data (Table 1). The GC contents of the two varieties were 43.65% and 43.78%. The Q30 percentages were 80.04% and 80.12%. The length distributions of the contigs, transcripts and unigenes are shown in Table 2, and using the Trinity de novo assembly program, the next-generation short-read sequences were assembled into 73,128 transcripts with a mean length of 978.92 bp. The transcripts were subjected to cluster and assembly analyses. A total of 42,881 unigenes with an average length of 787.30 bp were obtained, including 10,895 unigenes (25.41%) that were greater than 1 kb. The N50 values of the transcripts and unigenes were 1,552 bp and 1,304 bp, respectively.

**Table 1:** Output statistics of sequencing.

Sample	Total reads	Total nucleotides (bp)	GC percentage	Q30 percentage	Total mapping reads
BG	22,410,309	4,524,731,460	43.65%	80.04%	18,648,672 (83.21%)
GLBG	24,894,222	5,026,550,982	43.78%	80.12%	20,761,402 (83.40%)

**Table 2:** Length distribution of the assembled contigs, transcripts, and unigenes.

Nucleotide length (bp)	Contigs	Transcripts	Unigenes
200–300	3,005,034 (98.82%)	15,773 (21.57%)	12,836 (29.93%)
300–500	13,439 (0.44%)	15,087 (20.63%)	10,404 (24.26%)
500–1000	10,964 (0.36%)	16,728 (22.87%)	8,746 (20.40%)
1000–2000	8,275 (0.27%)	16,915 (23.13%)	7,538 (17.58%)
2000+	3,167 (0.10%)	8,625 (11.79%)	3,357 (7.83%)
Total number	3,040,879	73,128	42,881
Total length	157,416,545	71,586,356	33,760,304
N50 length	48	1,552	1,304
Mean length	51.77	978.92	787.30

The reads and unigenes were more than 83% similar in each sample (Table S1). As expected for a randomly fragmented transcriptome, the distribution curve for each sample was relatively flat (Figure S1). These results indicate that the throughput and sequencing quality were sufficient for the subsequent analyses.

### SSR Development and Analysis

SSRs are highly informative and are widely used in genetics, evolution and breeding studies. Approximately 3–7% of the expressed genes contain putative SSR motifs, mainly within the un-translated regions of the mRNA [40]. Using MISA, we identified 1,558 SSRs or microsatellites (Table S3) from 10,895 unigenes distributed in 1,374 sequences (Table 3), of which 151 sequences contained more than 1 SSR. Mononucleotide repeats were the most common form of microsatellites (796), followed by trinucleotide repeats (528) and dinucleotide repeats (216). Among the mononucleotide repeats, the A/T types were the most common repeats in the recovered unigenes (46.08%). TTC/GAA/CTT/GAG/CAT repeats accounted for 7.83% of the total number of repeats in the unigenes, and the most frequent dinucleotide repeats were AT/TA (4.75%).

**Table 3:** Summary of the simple sequence repeat (SSR) types.

Searched item	Numbers
Total number of examined sequences	10,895
Total number of identified SSRs	1,558
Number of SSR-containing sequences	1,374
Number of SSRs present in compound formation	62
Mono-nucleotides	796
Di-nucleotides	216
Tri-nucleotides	528
Tetra-nucleotides	16
Penta-nucleotides	2

### Functional Annotation and Classification

**Figure 2:**
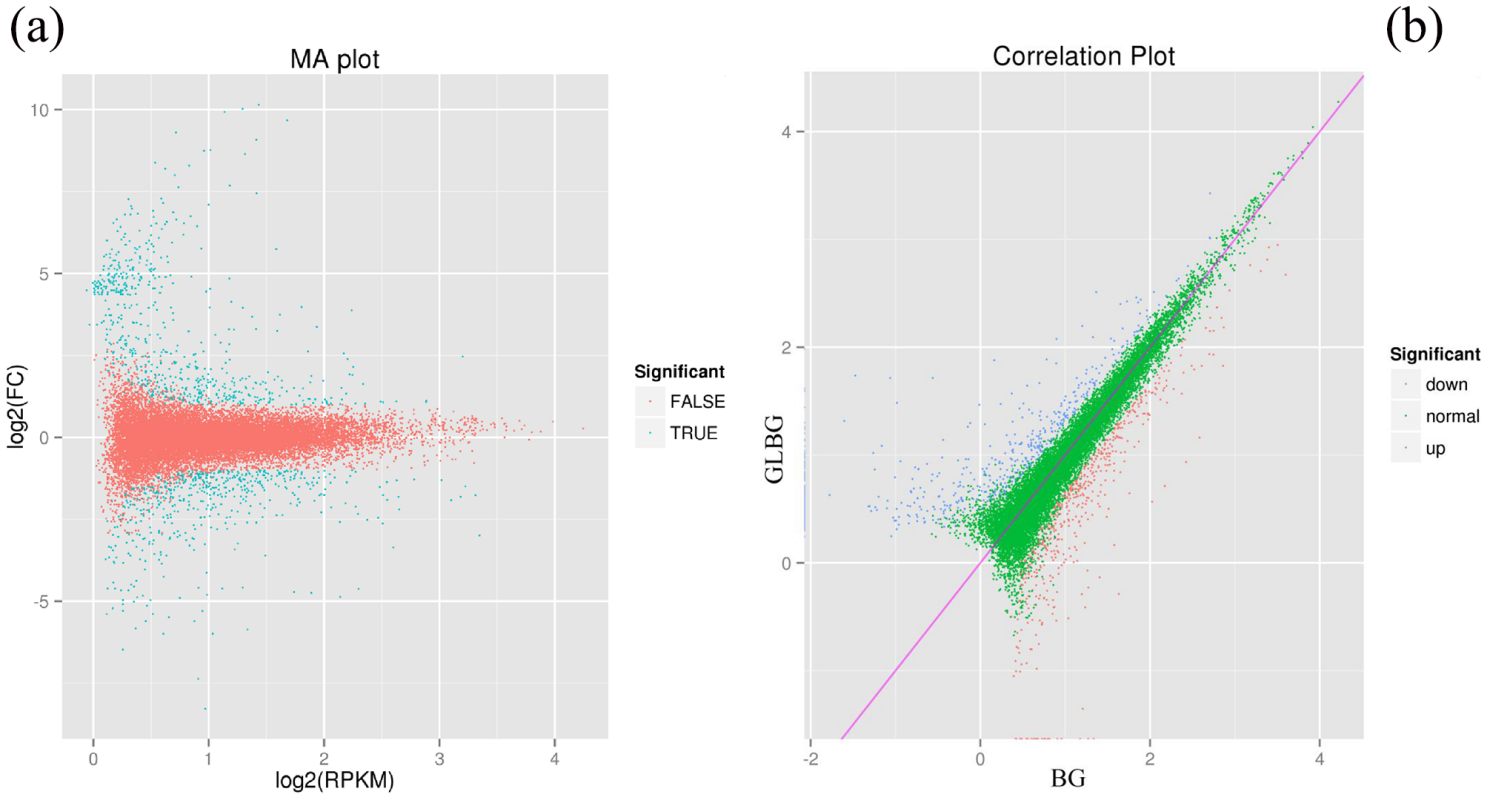
Differentially expressed genes of the cuticular wax biosynthesis. (a) Blue dots represent differentially expressed genes; red dots represent non-differentially expressed genes. (b) Blue dots represent the up-regulated DEGs; red dots represent the down-regulated DEGs. BG: a variety whose epidermal cells are covered by wax; GLBG: a variety whose epidermal cells are not covered by wax.

Among the 42,881 high-quality unigenes, 22,289 (52.0%) had at least one significant match to an existing gene model in BLAST searches (Table 4), and these unigenes were determined against the NCBI NR, Swiss-Prot, KEGG, GO, and COG databases using BLASTx to identify the proteins with the highest sequence similarity with the given unigenes and to determine their functional annotations. We screened differentially expressed genes (DEGs) and extracted identified information from the 22,289 unigenes; an overview of the sequencing statistical results is presented in Table 4 and Figure 2a. The up-regulated and down-regulated DEGs are shown in Figure 2b.

**Table 4:** Functional annotation of the unigenes.

Annotated database	Annotated number	300≤ length <1,000	Length ≧1,000
Nr Annotation	22,178	19,260	10,173
SwissProt Annotation	14,815	13,022	7,330
COG Annotation	7,428	6,903	4,569
GO Annotation	16,262	14,229	7,908
KEGG Annotation	5,308	4,702	2,745
All Annotations	22,289	19,332	10,177

**Figure 3:**
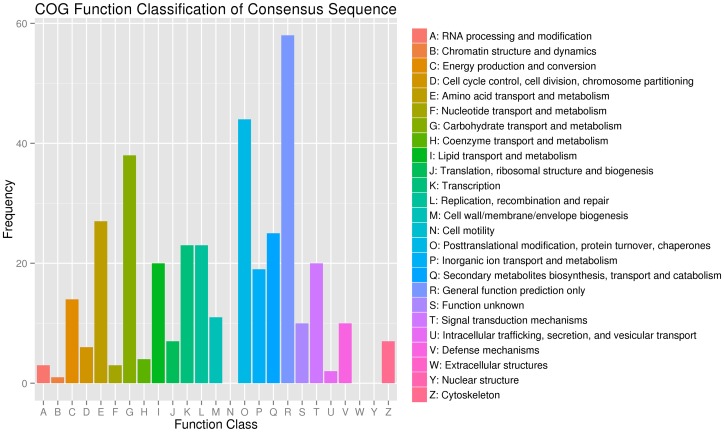
Cluster of orthologous groups (COG) classification. In total, 279 of the 7,428 sequences with Nr hits were grouped into 22 classifications.

In the COG functional classification, 279 of the 7,428 unigenes could be annotated in 22 COG categories (Figure 3). Among these unigenes, the general function prediction was the largest group (58), followed by posttranslational modification, protein turnover, and chaperones (44); carbohydrate transport and metabolism (38); amino acid transport and metabolism (27); secondary metabolite biosynthesis, transport and catabolism (25); transcription (23); and replication, recombination and repair (23).

### Gene Ontology (GO) Classification

GO terms are a dynamically structured control vocabulary that can be used to describe the functions of genes and by which genes can be classified into three major categories based on sequence homology, namely, biological process, molecular function, and cellular components, and their sub-categories [41]. There were 16,220 unigenes, of which 539 were grouped into 46 differentially expressed GO functional categories. Biological process (423) constituted the majority of the GO annotations, followed by molecular function (416) and cellular components (383).

**Figure 4:**
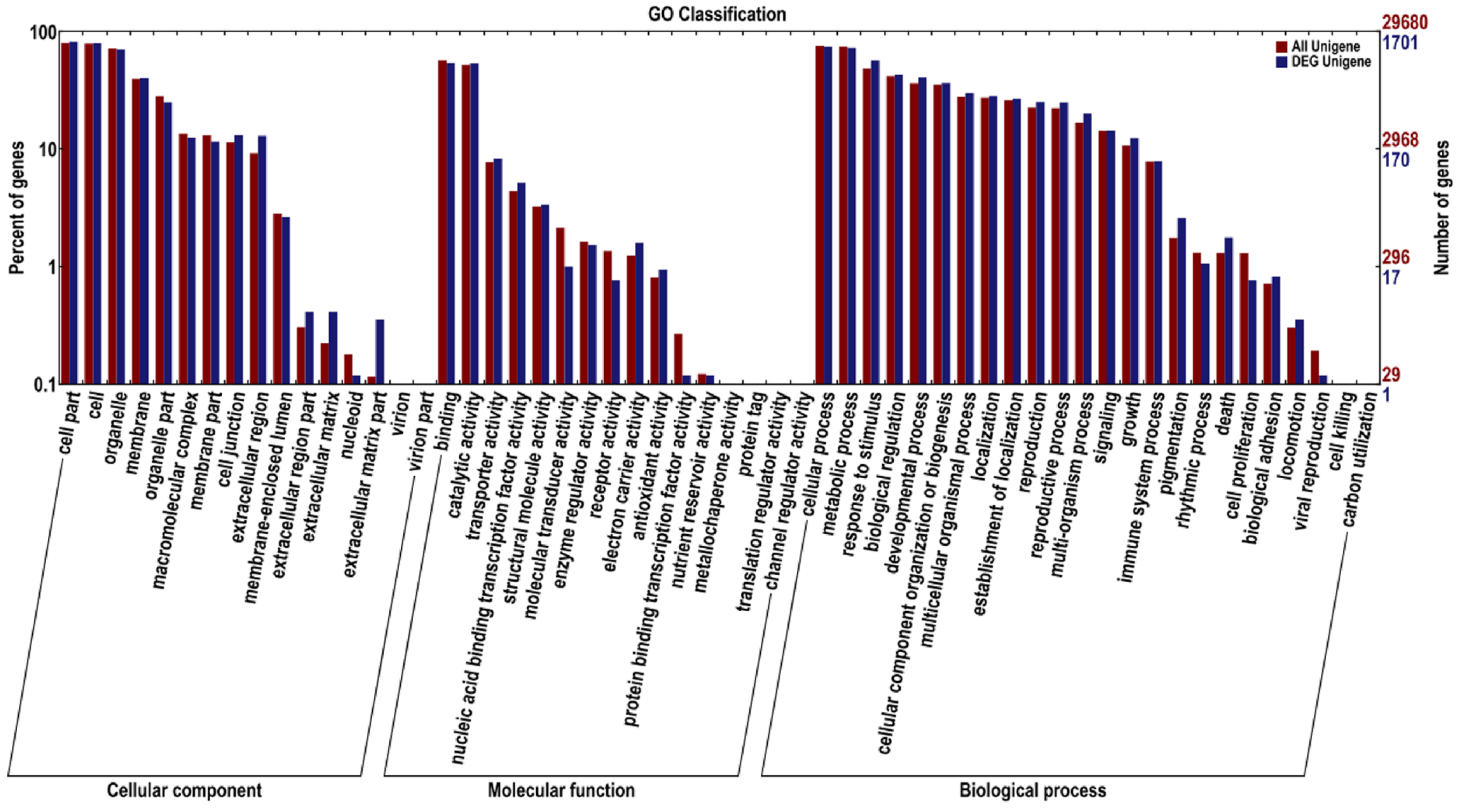
Functional annotation of assembled sequences based on gene ontology (GO) categorization. The unigenes are summarized into three main categories: cellular component, molecular function and biological process. The right side of the Y axis is the number of the genes; the left side of the Y axis is the percent of genes.

The major different sub-categories are shown in Figure 4. The three sub-categories “cell part” (GO: 0044464), “cell” (GO: 0005623) and “organelle” (GO: 0043226) were in the cluster of cellular components; in the cluster of molecular function were the two sub-categories “catalytic functions” (GO: 0003824) and “binding functions” (GO: 0005488); and the four sub-categories “metabolic process” (GO: 0008152), “cellular process” (GO: 0009987), “response to stimulus” (GO: 0050896), and “biological regulation” (GO: 0065007) were in the cluster of biological processes. However, there were no differences in the following sub-categories: extracellular region part and virion part in the cellular components; channel regulator activity, metallochaperone activity, protein tag, translation regulator activity and nutrient reservoir activity in molecular functions; and cell killing, carbon utilization, viral reproduction and rhythmic process in biological processes.

### Pathway Enrichment Analysis of DEGs

A KEGG pathway enrichment analysis was performed to categorize the biological functions of DEGs. We mapped all of the genes to terms in the KEGG database. The KEGG database can be used to categorize gene functions with an emphasis on biochemical pathways. To better understand the biological pathways in the Welsh onion, a BLASTx search against the KEGG protein database was performed using the assembled unigenes. A total of 102 unigenes were annotated with 66 pathways in the KEGG database. The predicted pathways represented the majority of plant biochemical pathways, including metabolism, genetic information processing, cellular processes, and organism systems. The pathways with the highest unigene representation were the starch and sucrose metabolism pathways (12.75%), followed by the galactose metabolism pathway (7.84%). Some pathways, such as the protein processing in endoplasmic reticulum pathways (6.86%), pentose and glucuronate interconversion pathways (5.88%), fatty acid biosynthesis pathways (4.90%), glycolysis/gluconeogenesis pathways (3.92%) and pyruvate metabolism pathways (3.92%), also had a significant portion of DEGs with pathway annotation. In the above highest unigene representation KEGG pathway, the enzymes α-galactosidase, pyruvate kinase, β-glucosidase and pectinesterase were related to down-regulated genes. Enzymes including tyrosine transaminase, polygalacturonase, UDP-glucuronate 4-epimerase, and Hsp70, Hsp90, and sHSF in ERAD were related to the up-regulated genes. 6-Phosphofructokinase and β-fructofuranosidase were related to both the up-regulated and the down-regulated genes.

### Genes Related to the Cuticular Wax Biosynthesis Pathway of DEGs

We identified 35 unigenes using BLAST that were associated with cuticular wax biosynthesis, of which four exhibited significantly differential expression levels in different pathways (Table 5). These four unigene IDs were comp35646, comp35656, comp35894 and comp54799, and they functioned in defense mechanisms and lipid transport and metabolism in the COG class annotation; they annotated as hypothetical protein (comp35646), fatty acid hydroxylase superfamily (comp35656), protein WAX2 isoform 1 (comp35894) and ABC transporter G family member (comp54799) in the Nr annotation; for the Swissprot annotation, they were involved in the long-chain acyl-CoA synthetase 2 (comp35646), protein ECERIFERUM 3 (comp35656 and comp35894) and ABC transporter G family member 12 (comp54799).

**Table 5:** The unigenes associated with cuticular wax biosynthesis.

Gene	Unigene ID	COG class annotation	Nr annotation	SwissProt annotation
1	comp35646	Lipid transport and metabolism	hypothetical protein	Long chain acyl-CoA synthetase 2
2	comp35656	Lipid transport and metabolism	Fatty acid hydroxylase superfamily	Protein ECERIFERUM 3
3	comp35894	Lipid transport and metabolism	protein WAX2 isoform 1	Protein ECERIFERUM 3
4	Comp54799	Defense mechanisms	ABC transporter G family member	ABC transporter G family member 12

These four unigenes were validated through RT-qPCR (Table 6); the transcript expression was down-regulated in non-waxy Welsh onion, which was verified with the RNA-seq dataset. The GO analysis demonstrated that these four unigenes had important functions in long-chain fatty acid metabolism and metabolism, wax biosynthesis, fatty acid biosynthesis, alkane biosynthesis, and other processes. The molecular functions of these unigenes included oxidoreductase activity, iron ion binding, nucleotide binding, ATP binding, organic phosphonate transmembrane-transporting ATPase activity, long-chain fatty acid-CoA ligase activity and very long-chain fatty acid-CoA ligase activity. The cellular functions of these unigenes included integral to the membrane, plasmodesma, nucleus, plasma membrane, and endoplasmic reticulum. However, there were no up-regulated DEGs in the genes related to the cuticular wax biosynthesis pathway.

**Table 6:** The candidate unigenes comparison result between qRT-PCR validation and RNA-Seq in Welsh onion transcriptome.

Unigene ID	2^-ΔΔ^CT(qRT-PCR valuation)		log_2_FC(RNA-Seq)	
	BG	GLBG	BG	GLBG
comp35646	1	0.358 (±0.00)	1	−5.014
comp35656	1	0.693 (±0.01)	1	−1.308
comp35894	1	0.665 (±0.01)	1	−1.556
Comp54799	1	0.555 (±0.01)	1	−1.319

BG: a variety covered by Wax on the epidermal cells; GLBG: a variety not covered by Wax on the epidermal cells. The candidate unigenes comparison result showed that the transcript expression was down-regulated in non-waxy Welsh onion (GLBG).

## Discussion

In recent years, transcriptome sequencing has become an effective tool used to discover molecular markers and identify novel genes. With the improvement of read length by paired-end sequencing, relatively short reads can be effectively assembled and have been successfully used to study plants without a genomic sequence [42],[43]. The study of Kolattu-kudy [44],[45] provides insights into the basic information necessary to analyze and identify the products that are synthesized by the waxy gene. Due to the paucity of studies on wax-related genes in the Welsh onion, the important waxy metabolism genes in the Welsh onion are not known. This study used the Illumina HiSeq 2000 platform for RNA-Seq to profile the Welsh onion transcriptome. In addition, the functions of the unigenes were classified by the COG and GO annotations and the metabolic pathways. A total of 798 genes, representing 1.86% total putative unigenes, were differentially expressed between the waxy Welsh onion and non-waxy mutant Welsh onion varieties.

Through SwissProt annotation, four important waxy synthetic unigenes of Welsh onion were found, and the results showed that the waxy synthesis protein might be controlled by Protein ECERIFERUM 3, Long-chain acyl-CoA synthetase 2 (lacs2) waxy gene synthesis in lipid transport and metabolism, and the ABC transporter G family member 12 in the defense mechanism. Lacs2 is encoded by a gene family that consists of at least nine genes in Arabidopsis (*Arabidopsis thaliana*) coding for enzymes that function in lipid synthesis, fatty acid catabolism, and the transport of fatty acids between subcellular compartments. Schnurr [46] observed lacs2 gene expression in the fast-growing young tissue, but in leaves, the expression is confined to the near- and far-shaft axis epidermis cells. The wax ingredients in the waxy leaf surface are similar to those of non-wax leaves, but the total wax load on non-waxy leaves was greater than that on waxy leaves; therefore, the epidermis cuticle thickness of waxy leaves decreased. Todd [47] cloned the kscI gene, which encodes 3-ketoacyl-CoA synthase; this enzyme is involved in the synthesis of long-chain fatty acids and wax in plant tissues. C26 to C30 wax alcohols and wax aldehydes decrease by more than 80% when kscI gene expression is suppressed. Chen [48] cloned and identified *wax2* from *Arabidopsis thaliana*, which can affect both the composition and structure of the waxy layer of the epidermis and the horny layer, and predicted that *wax2* has a metabolic function that is associated with both the cuticle membrane and wax synthesis in Arabidopsis. These results demonstrate that the four unigenes identified in the present study are closely related to waxy cuticle synthesis and are down-regulated 1- to 3-fold in non-waxy leaves, as verified with RNA-seq. This study confirms that the four unigenes comp35646, comp35656, comp35894 and comp54799 are important wax-related genes in Welsh onion and that the expression of these genes correlates with the wax content in Welsh onion.

**Figure 5:**
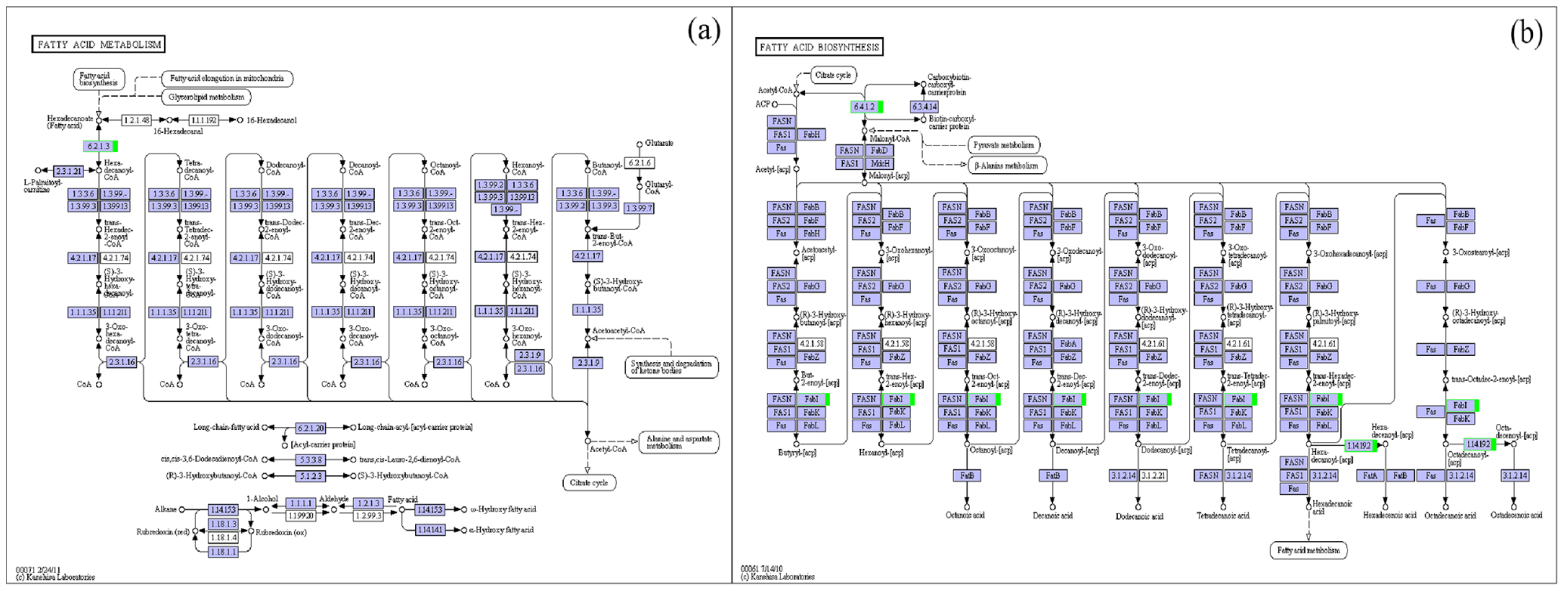
Welsh onion unigenes involved in two secondary metabolic pathways KEGG pathways. (a) Fatty acid metabolism; (b) fatty acid biosynthesis. Blue boxes: this enzyme specific to this species; Green boxes: down-regulated genes.

The KEGG database can be used to categorize gene functions with an emphasis on biochemical pathways. For all of the plants in this study, the enzymatic formation of waxy synthetic precursor VLCFAs occurred on the endoplasmic reticulum. Our research on the GO annotation of four Welsh onion waxy genes indicates that these genes function in wax biosynthesis and that the endoplasmic reticulum integrated with the membrane and plasmodesma in cellular components may play an important role in wax synthesis, indicating that the entire process from VLCFAs to the final synthesis of waxy components occurs in the endoplasmic reticulum. The waxy product must be transported from the endoplasmic reticulum to the plasma membrane and then to the plant cell wall through the epidermis. Wax transportation from the hydrophobic membrane lipid bilayer to the hydrophilic apoplast is an energy-intensive process. The ABC transporter protein in our research may be involved in this process [49],[50]. This ABC transporter protein is homologous to a known lipid transfer protein, is positioned on the membrane, and can provide energy through the hydrolysis of ATP, indicating that this protein might play a role in wax secretion. Because the fatty acid cycle plays an important role in waxy cuticle synthesis, our research indicates that in the fatty acid biosynthesis and fatty acid metabolism KEGG pathway (Figure 5), relative to the reference group, the enzymes long-chain-fatty-acid-CoA ligase, acetyl-CoA carboxylase (ACCase), acyl-[acyl-carrier-protein] desaturase and 1-phosphatidylinositol-3-phosphate 5-kinase were down-regulated in the experimental group whose epidermal cells were not covered by wax. ACCase catalyzes the ATP-dependent carboxylation of acetyl-CoA to form malonyl-CoA, which is the first and committing step in de novo fatty acid biosynthesis [51]. Amid et al. [52] cloned the sfr3 gene, a missense mutation in ACC1, which is an essential gene encoding homomeric (multifunctional) ACCase. The wax deposition on the inflorescence stem of cold-grown sfr3 plants was inhibited and the long-chain components of the leaf cuticular wax were reduced compared to those of the wild-type plants. Therefore, the change in these enzymes might suppress waxy cuticle synthesis.

This study used RNA-seq to investigate waxy cuticle-related genes and pathways in Welsh onion. We found four important unigenes relate to the wax content in Welsh onion and developed 1,558 SSR molecular markers with which to construct a high-resolution genetic map in the future. We believe that this transcriptome dataset will accelerate the research on waxy gene clones and other functional genomics research on *Allium fistulosum*.

## Supporting Information

Figure S1 (a) Randomness test of cDNA fragments; (b) Sequencing saturation analysis.
(TIF)

Table S1 **Statistical comparison sample sequencing data.**
(DOCX)

Table S2 **Primers used to perform the qPCR of waxy cuticle-related gene biosynthesis and regulated genes.**
(DOCX)

Table S3 **SSR sequencing data.**
(XLS)

## References

[1] Ueda H, Takeuchi A, Wako T (2013) Activation of Immune Responses in Mice by an Oral Administration of Bunching Onion (*Allium fistulosum*) Mucus. Bioscience, biotechnology, and biochemistry 77(9):1809–1813. 10.1271/bbb.130084 24018671

[2] Jiangsu New Medical College (1986) Dictionary of traditional Chinese Medicine. Shanghai Science and Technology, Publisher, Shanghai, 2316–2318.

[3] Tawaraya K, Hirose R, Wagatsuma T (2012) Inoculation of arbuscular mycorrhizal fungi can substantially reduce phosphate fertilizer application to *Allium fistulosum* L. and achieve marketable yield under field condition. Biology and Fertility of Soils 48(7):839–843.

[4] Nejkov S, Ovcharova A, Babrikov T (2013) Investigation of the Relationship Irrigation–Yield for the Cultivars of Welsh Onion *Allium fistulosum* L. Растениевъдни Науки (България). 50(1):78–82.

[5] Dong YX, Cheng ZH, Meng HW, Liu HQ, Wu CN, et al (2013) The effect of cultivar, sowing date and transplant location in field on bolting of welsh onion (*Allium fistulosum* L.). BMC plant biology 13(1):154. 24199907 10.1186/1471-2229-13-154 PMC4226261

[6] Tanino KK, Kobayashi S, Hyett C, Hamilton K, Liu J, et al (2013) *Allium fistulosum* as a novel system to investigate mechanisms of freezing resistance. Physiologia Plantarum 147(1):101–111. 23078395 10.1111/j.1399-3054.2012.01716.x

[7] Vu HQ, El-Sayed MA, Ito SI, Yamauchi N. ShigyoacM (2012) Discovery of a new source of resistance to *Fusarium oxysporum*, cause of *Fusarium* wilt in *Allium fistulosum*, located on chromosome 2 of *Allium cepa* Aggregatum group. Genome 55(10):797–807. 23199574 10.1139/g2012-065

[8] Xu N, Wei M, Wang C, Shi W, Tian F, et al (2013) Composition of Welsh onion (*Allium fistulosum* L.) root exudates and their allelopathy on cucumber sprouts and Fusarium oxysporum f. sp. cucumerinum. Allelopathy Journal 32(2):243–256.

[9] Sung YY, Yoon T, Kim SJ, Yang WK, Kim HK (2011) Anti-obesity activity of *Allium fistulosum* L. extract by down-regulation of the expression of lipogenic genes in high-fat diet-induced obese mice. Molecular Medicine Reports 4:431–435. 21468588 10.3892/mmr.2011.451

[10] Ueda H, Takeuchi A, Wako T (2013) Activation of Immune Responses in Mice by an Oral Administration of Bunching Onion (*Allium fistulosum*) Mucus. Bioscience, biotechnology, and biochemistry 77(9):1809–13. 10.1271/bbb.130084 24018671

[11] Tsukazaki H, Yaguchi S, Yamashita K, Shigyo M, Kojima A, et al (2012) QTL analysis for pseudostem pungency in bunching onion (*Allium fistulosum*). Molecular Breeding 30(4):1689–1698.

[12] Kunst L, Samuels AL (2003) Biosynthesis and secretion of plant cuticular wax. Progress in Lipid Research 42(1):51–80. 12467640 10.1016/s0163-7827(02)00045-0

[13] Riederer M, Schneider G (1990) The effect of the environment on the permeability and composition of *Citrus* leaf cuticles. Planta 180(2):154–165. 24201939 10.1007/BF00193990

[14] Jenks MA, Joly RJ, Peters PJ, Rich PJ, Axtell JD, et al (1994) Chemically induced cuticle mutation affecting epidermal conductance to water vapor and disease susceptibility in Sorghum bicolor (L.) Moench. Plant Physiology 105(4):1239–1245. 12232280 10.1104/pp.105.4.1239 PMC159454

[15] Kolattukudy P E (1996) Biosynthetic pathways of cutin and waxes, and their sensitivity to environmental stresses. Plant cuticles: an integrated functional approach. BIOS Scientific Publishers, Oxford, 83–108.

[16] Krauss P, Markstädter C, Riederer M (1997) Attenuation of UV radiation by plant cuticles from woody species. Plant, Cell & Environment 20(8):1079–1085.

[17] Sieber P, Schorderet M, Ryser U, Buchala A, Kolattukudy P, et al (2000) Transgenic Arabidopsis plants expressing a fungal cutinase show alterations in the structure and properties of the cuticle and postgenital organ fusions. The Plant Cell 12(5):721–737. 10810146 10.1105/tpc.12.5.721 PMC139923

[18] Kurata T, Kawabata-Awai C, Sakuradani E, Shimizu S, Okada K, et al (2003) The *YORE-YORE* gene regulates multiple aspects of epidermal cell differentiation in *Arabidopsis* . The Plant Journal 36(1):55–66. 12974811 10.1046/j.1365-313x.2003.01854.x

[19] James DW Jr, Lim E, Keller J, Plooy I, Ralston E, et al (1995) Directed tagging of the Arabidopsis *FATTY ACID ELONGATION1(FAE1)* gene with the maize transposon activator. The Plant Cell 7(3):309–319. 7734965 10.1105/tpc.7.3.309 PMC160784

[20] Millar AA, Clemens S, Zachgo S, Giblin M, Taylor DC, et al (1999) *CUT1*, an Arabidopsis gene required for cuticular wax biosynthesis and pollen fertility, encodes a very-long-chain fatty acid condensing enzyme. The Plant Cell 11(5):825–838. 10330468 10.1105/tpc.11.5.825 PMC144219

[21] Todd J, Post-Beittenmiller D, Jaworski JG (1999) *KCS1* encodes a fatty acid elongase 3-ketoacyl-CoA synthase affecting wax biosynthesis in *Arabidopsis thaliana* . Plant Journal 17(2):119–130. 10074711 10.1046/j.1365-313x.1999.00352.x

[22] Fiebig A, Mayfield JA, Miley NL, Chau S, Fischer RL, et al (2000) Alterations in *CER6*, a gene identical to *CUT1*, differentially affect long-chain lipid content on the surface of pollen and stems. The Plant Cell 12(10):2001–2008. 11041893 10.1105/tpc.12.10.2001 PMC149136

[23] Pruitt RE, Vielle-Calzada JP, Ploense SE, Grossniklaus U, Lolle S, et al (2000) *FIDDLEHEAD*, a gene required to suppress epidermal cell interactions in *Arabidopsis*, encodes a putative lipid biosynthetic enzyme. Proceedings of the National Academy of Sciences 97(3):1311–1316. 10.1073/pnas.97.3.1311 PMC15605 10655527

[24] Xu XJ, Dietrich CR, Lessire R, Nikolau BJ, Schnable PS (2002) The endoplasmic reticulum-associated maize GL8 protein is a component of the acyl-coenzyme A elongase involved in the production of cuticular waxes. Plant Physiology 128(3):924–934. 11891248 10.1104/pp.010621 PMC152205

[25] Dietrich CR, Perera MA, DYandeau-Nelson M, Meeley RB, Nikolau BJ, et al (2005) Characterization of two GL8 paralogs reveals that the 3-ketoacyl reductase component of fatty acid elongase is essential for maize (Zea maysL.) development. Plant Journal 42(6):844–861. 15941398 10.1111/j.1365-313X.2005.02418.x

[26] Zheng H, Rowland O, Kunst L (2005) Disruptions of theArabidopsis Enoyl-CoA reductase gene reveal an essential role for very-long-chain fatty acid synthesis in cell expansion during plant morphogenesis. The Plant Cell 17(5):1467–1481. 15829606 10.1105/tpc.104.030155 PMC1091768

[27] Bach L, Michaelson LV, Haslam R, Bellec Y, Gissot L, et al (2008) The very-long-chain hydroxy fatty acyl-CoA dehydratase PASTICCINO2 is essential and limiting for plant development. Proceedings of the National Academy of Sciences 105(38):14727–14731. 10.1073/pnas.0805089105 PMC2567193 18799749

[28] Rowland O, Zheng H, Hepworth SR, Lam P, Jetter R, et al (2006) *CER4* encodes an alcohol-forming fatty acyl-coenzyme: A reductase involved in cuticular wax production in Arabidopsis. Plant Physiology 142(3):866–877. 16980563 10.1104/pp.106.086785 PMC1630741

[29] Li F, Wu X, Lam P, Bird D, Zheng H, et al (2008) Identification of the wax ester synthase/acyl-coenzyme A: diacylglycerol acyltransferase WSD1 required for stem wax ester biosynthesis in Arabidopsis. Plant Physiology l148(1):97–107. 10.1104/pp.108.123471 PMC2528131 18621978

[30] Greer S, Wen M, Bird D, Wu X, Samuels L, et al (2007) The cytochrome P450 enzyme CYP96A15 is the midchain alkane hydroxylase responsible for formation of secondary alcohols and ketones in stem cuticular wax of Arabidopsis. Plant Physiology 145(3):653–667. 17905869 10.1104/pp.107.107300 PMC2048791

[31] Pighin JA, Zheng H, Balakshin LJ, Goodman LP, Western TL, et al (2004) Plant cuticular lipid export requires an ABC transporter. Science 306(5696):702–704. 15499022 10.1126/science.1102331

[32] Shi CY, Yang H, Wei CL, Yu O, Zhang ZZ, et al (2011) Deep sequencing of the Camellia sinensis transcriptome revealed candidate genes for major metabolic pathways of tea-specific compounds. BMC genomics 12(1):131. 21356090 10.1186/1471-2164-12-131 PMC3056800

[33] Grabherr MG, Haas BJ, Yassour M, Levin JZ, Thompson DA, et al (2011) Full-length transcriptome assembly from RNA-Seq data without a reference genome. Nature Biotechnology 29:644–652. 10.1038/nbt.1883 PMC3571712 21572440

[34] Liu M, Qiao G, Jiang J, Yang H, Xie J, et al (2012) Transcriptome sequencing and *de novo* analysis for ma bamboo (*Dendrocalamus latiflorus* Munro) using the Illumina platform. Plos One 7(10):e46766. 23056442 10.1371/journal.pone.0046766 PMC3463524

[35] Langmead B, Trapnell C, Pop M, Salzberg SL (2009) Ultrafast and memory-efficient alignment of short DNA sequences to the human genome. Genome Biology 10(3):R25. 19261174 10.1186/gb-2009-10-3-r25 PMC2690996

[36] Mortazavi A, Williams BA, McCue K, Schaeffer L, Wold B (2008) Mapping and quantifying mammalian transcriptomes by RNA-Seq. Nature Methods 5(7):621–628. 18516045 10.1038/nmeth.1226 PMC13303166

[37] Dai H, Han G, Yan Y, Zhang F, Liu Z, et al (2013) Transcript assembly and quantification by RNA-Seq reveals differentially expressed genes between Soft-Endocarp and Hard-Endocarp Hawthorns. Plos One 8(9):e72910. 24039819 10.1371/journal.pone.0072910 PMC3764154

[38] Conesa A, Götz S, García-Gómez J M, Terol J, Talón M, et al (2005) Blast2GO: a universal tool for annotation, visualization and analysis in functional genomics research. Bioinformatics 21(18):3674–3676. 16081474 10.1093/bioinformatics/bti610

[39] Romualdi C, Bortoluzzi S, d'Alessi F, Danieli GA (2003) IDEG6: a web tool for detection of differentially expressed genes in multiple tag sampling experiments. Physiological genomics 12(2):159–162. 12429865 10.1152/physiolgenomics.00096.2002

[40] Thiel T, Michalek W, Varshney RK, Graner A (2003) Exploiting EST databasesfor the development and characterization of gene-derived SSR-markers in barley (*Hordeum vulgare* L.). Theor Appl Genet 106(3):411–422. 12589540 10.1007/s00122-002-1031-0

[41] Ashburner M, Ball CA, Blake JA, Botstein D, Butler H, et al (2000) Gene Ontology: tool for the unification of biology. Nature Genetics 25(1):25–29. 10802651 10.1038/75556 PMC3037419

[42] Zhang J, Liang S, Duan J, Wang J, Chen S, et al (2012) De novo assembly and Characterisation of the Transcriptome during seed development, and generation of genic-SSR markers in Peanut (*Arachis hypogaea* L.). BMC genomics 13(1):90. 22409576 10.1186/1471-2164-13-90 PMC3350410

[43] Wei W, Qi X, Wang L, Zhang Y, Hua W, et al (2011) Characterization of the sesame (*Sesamum indicum* L.) global transcriptome using Illumina paired-end sequencing and development of EST-SSR markers. BMC genomics 12(1):451. 21929789 10.1186/1471-2164-12-451 PMC3184296

[44] Kolattukudy P E (1980) Biopolyester membranes of plants: cutin and suberin. Science 208(4447):990–1000. 17779010 10.1126/science.208.4447.990

[45] Kolattukudy P E (1981) Structure, biosynthesis, and biodegradation of cutin and suberin. Annual Review of Plant Physiology 32(1):539–567.

[46] Schnurr J, Shockey J (2004) The acyl-CoA synthetase encoded by *LACS2* is essential for normal cuticle development in Arabidopsis. The Plant Cell 16(3):629–642. 14973169 10.1105/tpc.017608 PMC385277

[47] Todd J, Post-Beittenmiller D, Jaworski J G (1999) *KCS1*encodes a fatty acid elongase 3-ketoacyl-CoA synthase affecting wax biosynthesis in *Arabidopsis thaliana* . The Plant Journal 17(2):119–130. 10074711 10.1046/j.1365-313x.1999.00352.x

[48] Chen X, Goodwin SM, Boroff VL, Liu X, Jenks MA (2003) Cloning and characterization of the *WAX2* gene of Arabidopsis involved in cuticle membrane and wax production. The Plant Cell 15(5):1170–1185. 12724542 10.1105/tpc.010926 PMC153724

[49] Qin P, Tu B, Wang Y, Deng L, Quilichini TD, et al (2013) *ABCG15* encodes an ABC transporter protein, and is essential for post-meiotic anther and pollen exine development in Rice. Plant and Cell Physiology 54(1):138–154. 23220695 10.1093/pcp/pcs162

[50] Pighin J A, Zheng H, Balakshin LJ, Goodman IP, Western TL, et al (2004) Plant cuticular lipid export requires an ABC transporter. Science 306(5696):702–704. 15499022 10.1126/science.1102331

[51] Nikolau BJ, Ohlrogge JB, Wurtele ES (2003) Plant biotin-containing carboxylases. Archives of Biochemistry and Biophysics 414(2):211–222. 12781773 10.1016/s0003-9861(03)00156-5

[52] Amid A, Lytovchenko A, Fernie AR, Warren G, Thorlby GJ (2012) The *sensitive* to *freezing3* mutation of *Arabidopsis thaliana* is a cold-sensitive allele of homomeric acetyl-CoA carboxylase that results in cold-induced cuticle deficiencies. Journal of Experimental Botany 63(14):5289–5299. 22791831 10.1093/jxb/ers191 PMC3431002

