# Supplementary figures and images for: RNA-Seq Reveals Leaf Cuticular Wax-Related Genes in Welsh Onion
Source: PLoS One. 2014 Nov 21;9(11):e113290. doi: 10.1371/journal.pone.0113290 (PMC4240658; doi:10.1371/journal.pone.0113290)

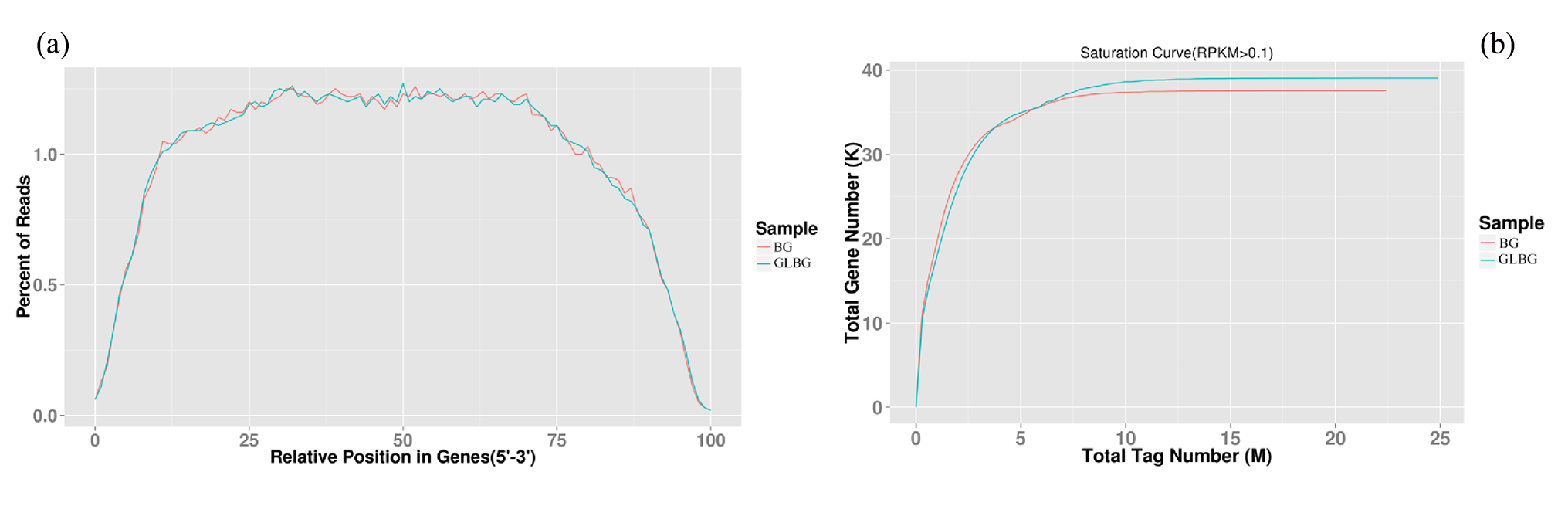

Supplement: Figure S1 — (a) Randomness test of cDNA fragments; (b) Sequencing saturation analysis. (TIF) [file pone.0113290.s001.tif]
