# Supplementary material for: RNA-Seq Reveals Leaf Cuticular Wax-Related Genes in Welsh Onion
Source: PLoS One. 2014 Nov 21;9(11):e113290. doi: 10.1371/journal.pone.0113290 (PMC4240658; doi:10.1371/journal.pone.0113290)
Supplement: Table S1 — Statistical comparison sample sequencing data. (DOCX) [file pone.0113290.s002.docx]

Table S1. Statistical comparison sample sequencing data.

| ID | Total reads | Total mapping reads | Uniquely mapped  reads | Multiply mapped  reads |
| --- | --- | --- | --- | --- |
| BG | 22,410,309 | 18,648,672 (83.21%) | 14,724,635 (78.96%) | 3,924,037 (21.04%) |
| GLBG | 24,894,222 | 20,761,402 (83.40%) | 16,485,500 (79.40%) | 4,275,902 (20.60%) |
