# Supplementary material for: RNA-Seq Reveals Leaf Cuticular Wax-Related Genes in Welsh Onion
Source: PLoS One. 2014 Nov 21;9(11):e113290. doi: 10.1371/journal.pone.0113290 (PMC4240658; doi:10.1371/journal.pone.0113290)
Supplement: Table S2 — Primers used to perform the qPCR of waxy cuticle-related gene biosynthesis and regulated genes. (DOCX) [file pone.0113290.s003.docx]

| Table S2. Primers used to perform the qPCR of waxy cuticle-related gene biosynthesis and regulated genes. | |
| --- | --- |
| Unigene ID | Primer sequence (5’ to 3’) |
| comp35646 | Forward: GATGCGTAAGTCGTCCAAATG |
|  | Reverse: GAACCACCTTCTGGGATTGA |
| comp35656 | Forward: CAGCCTACATCACAGCGAAA |
|  | Reverse: AGGAACACGAAATCAGGAACC |
| comp35894 | Forward: CAGCCTACATCACAGCGAAA |
|  | Reverse: CCAGGAACACGAAATCAGGA |
| comp54799 | Forward: CTGTGATTTCCTCGGTTCATC |
|  | Reverse: CTTAGTAGCCAGTTTGGAGTCG |
| Actin | Forward: ACACGGCCTGGATAGCAACAT |
|  | Reverse: AGAGCAGTATTCCCAAGCATT |
